# The nature of thinking, shallow and deep

**DOI:** 10.3389/fpsyg.2014.00435

**Published:** 2014-05-15

**Authors:** Gary L. Brase

**Affiliations:** Department of Psychological Sciences, Kansas State UniversityManhattan, KS, USA

**Keywords:** normative models, cognitive modularity, deep rationality, evolutionary psychology, human reasoning, time scales in rational decision making

## Abstract

Because the criteria for success differ across various domains of life, no single normative standard will ever work for all types of thinking. One method for dealing with this apparent dilemma is to propose that the mind is made up of a large number of specialized modules. This review describes how this multi-modular framework for the mind overcomes several critical conceptual and theoretical challenges to our understanding of human thinking, and hopefully clarifies what are (and are not) some of the implications based on this framework. In particular, an evolutionarily informed “deep rationality” conception of human thinking can guide psychological research out of clusters of *ad hoc* models which currently occupy some fields. First, the idea of deep rationality helps theoretical frameworks in terms of orienting themselves with regard to time scale references, which can alter the nature of rationality assessments. Second, the functional domains of deep rationality can be hypothesized (non-exhaustively) to include the areas of self-protection, status, affiliation, mate acquisition, mate retention, kin care, and disease avoidance. Thus, although there is no single normative standard of rationality across all of human cognition, there are sensible and objective standards by which we can evaluate multiple, fundamental, domain-specific motives underlying human cognition and behavior. This review concludes with two examples to illustrate the implications of this framework. The first example, decisions about having a child, illustrates how competing models can be understood by realizing that different fundamental motives guiding people’s thinking can sometimes be in conflict. The second example is that of personifications within modern financial markets (e.g., in the form of corporations), which are entities specifically constructed to have just one fundamental motive. This single focus is the source of both the strengths and flaws in how such entities behave.

## INTRODUCTION

It has been said that man is a rational animal. All my life I have been searching for evidence which could support this.

Russell (1950)

A foundational principle of science is that good scientific theories must generate testable, and therefore falsifiable, hypotheses. Research can then obtain data relevant to that hypothesis and show that the prediction either holds up or fails. Science thus has normative standards as an intrinsic property of the scientific method; the nature of good scientific theories includes the ability to provide testable predictions. Those predictions provide a standard for evaluating a theory, saying what ought to happen if the theory is correct. In this sense, then, normative models are an essential property of research on human though (or research on anything else). Certainly people can become confused between theoretical predictions of what “ought” to happen (in the sense of as the theory predicts) versus notions of “ought” based on a cultural or socio-moral position. That, however, is not a problem with normative standards as a property of scientific theories as much as it is a problem of people not understanding how science works. So, for example, when one asks “what *ought* human thinking be like?” it is important to clarify if the question is about a prediction based on a scientific theory or if the question carries some presumption of the inquisitor based on their personal views. One of these is science; the other is personal opinion.

The problem with normative models in the scientific study of human thought is that no single normative standard works for all types of thinking. How do we decide on appropriate normative standards? (Which, in this scientific sense means how do we decide upon appropriate theoretical frameworks?) Thinking is a ubiquitous feature of human activity, but the normative standards for evaluating good food are different from the normative standards for evaluating a good place to live, which are different from the normative standards for evaluating a good relationship partner, which are in turn different from the normative standards for evaluating a good stock market decision. In general terms, for any problem or task domain there is a set of features that define that problem/task and therefore those same features provide criteria for success (i.e., the “good” solution). The more one knows about the nature of the features that constitute a problem, the more one therefore knows about properties that can be exploited to get to an effective and efficient solution. For instance, some of the defining features of the problem of food acquisition are identifying high calorie, digestible items. The criteria for success (“good” food) are things which contain fats, sugars, carbohydrates, and proteins. Other things (dirt, wood, metal, plastic) do not satisfy these criteria. If one attempts to collapse across multiple problems or tasks to achieve a general-purpose solution method, the features that define the overall problem become increasingly general and computationally ineffectual. At moderate levels of generality we find problem solving tools that are simply weak (e.g., General Problem Solver; Newell and Simon, 1972). With further levels of generality we find only normative standard that are uselessly vague (“Don’t screw up.”) and computational incapacitation as a result.

Because different normative models provide standards of evaluation for different types, or domains, of behaviors, one of the key questions then is how to parse the various aspects of the world into domains. In which domains do which particular normative models apply? Some people will recognize this as the dilemma posed by the idea (from Plato’s *Phaedrus*) that scientific theories should “carve nature at its joints,” but the problem is that there does not appear to be any single carving pattern that consistently and uniquely works. Instead there seem to be multiple carving patterns that can each be legitimately argued for and that each nonetheless have flaws. In other words, even within a particular domain there are often multiple normative models which could be applied, and obeying one standard for rationality tends to lead to violations within other standards of rationality.

One method for dealing with the apparent dilemma is to propose that the mind is made up of a large number of specialized cognitive mechanisms, often referred to as “modules,” which each embody their own internal standards of correct solutions within that particular domain. This is often identified as an evolutionary approach, although the same conclusion can be obtained via other routes (for example, via functional neuroanatomical evidence). One can similarly reach this conclusion by considering the implications of combinatorial explosion when trying to program problem solving machinery in artificial intelligence (i.e., the “frame problem”), which has been identified in philosophy as the problem of indeterminacy in inference (Quine, 1960; Dennett, 1978, 1984). It is also increasingly a commitment required to make sense of the precocious abilities of infants when tested using means such as the habituation paradigm (e.g., Wynn, 1992; Hirschfeld and Gelman, 1994; Wagner and Carey, 2003; McCrink and Wynn, 2004; Xu et al., 2004). The cognitive development field often refers to this situation as the existence of “constraints” in human infant mental abilities, reflecting the default assumption of a completely domain-general and content independent cognitive architecture. These constraints, however, are actually the *enablers* of specific cognitive abilities because the particular skills which they shunt infants into developing would not be able to emerge without the guidance of those constraints.

Although various people fret about this proposal being “massive modularity” (Samuels, 1998) or “modularity gone mad” (Fodor, 1987), it is the conclusion which the evidence impels us to accept. Besides indications that modularity is inevitable based on logical principles (Cosmides and Tooby, 1994; Callebaut and Rasskin-Gutman, 2005; Tooby and Cosmides, 2005; Carruthers, 2006; Ermer et al., 2007), computer simulations show that modularity is a consequence of neural organization under realistic conditions (Bullinaria, 2006; Clune et al., 2013), and actual physical and neurological structures point empirically to modular organization (Geary and Huffman, 2002; Cheverud et al., 2007). There is a functional carving of mental abilities, and it is a relatively fine-grained carving compared to what has generally been considered before (e.g., Inhelder and Piaget, 1958; Newell and Simon, 1972; Kahneman and Tversky, 1979; Johnson-Laird and Byrne, 1991). Furthermore, these lines of evidence do not commit anyone to impose some of the properties of modularity suggested by early ideas (Fodor, 1983). Functionally specialized cognitive modules are not required to be informationally encapulated, or to accept only highly local inputs, or to be reflexive and insensitive to contexts (see Barrett, 2005; Barrett and Kurzban, 2006 for in depth discussions of how and why these properties are not required elements). Some modules may in fact have these properties, but that does not mean that all modules must. In other words, the joints of nature may be carvable, but the lines are not necessarily “clean.” Consider, by analogy, the various systems within the rest of the human body: respiration, digestion, circulation, etc. Many of these systems are intertwined, receiving inputs from each other and sending their outputs to other systems. Yet we still find it useful to separate these systems out for purposes of understanding and explaining them, and we can see the overall pattern of major functional adaptations embodied by these systems.

## DO WE *REALLY* NEED TO CHANGE?

A skeptical reader might ask, “But, these are theoretical issues about the grand nature of the entire human mind (or all thinking); do I really need to change at the level my actual research? That is, what is my concern so long as I stick to my particular topic?” The response is that these issues of the grand nature of human thinking can and do percolate down to specific research topics. Attending to them opens up opportunities, and neglecting them creates problems.

Consider the area of human reasoning, a topic and field that is central to the idea of “thinking,” and the most commonly used research tool in that field: Wason’s selection task. The selection task was originally devised by Wason (1966) to evaluate if people can engage in logical falsification as part of, for example, scientific hypothesis evaluation. The task involves presenting a conditional rule (of the form, *If P, then Q,* where *P* and *Q* can be any content), usually some contextual information for the rule, and then four pictures of cards which are described as having relevant information printed on both sides of them. The visible sides of the four cards provide information about all four possible states relevant to the rule: *P*, *not-P*, *Q*, and *not-Q*. The task for participants is to indicate which of the cards need to be turned over for further information in order to evaluate the validity of the conditional rule. So, for instance, turning over the *P* card would reveal information (either *Q* or *not-Q*), and this is information which bears on the truth or falsity of the conditional rule.

The most traditional normative model for the selection task is first order conditional logic. Given a rule of the form “If *P*, then *Q*” (again, where *P* and *Q* are any content whatsoever), there are logical conclusions that can be derived from additional pieces of information: if *P* is true, then *Q* is true; if *Q* is false (*not-Q*), then *P* is false (*not-P*). The cards which need to turned over for more information, according to formal logic, are the *P* and the *Not-Q* cards then, in order to assess if there are any violations of the rule. The general findings from Wason’s original work and many subsequent studies is that people are notoriously poor at logical falsification such as this, even though it is computationally quite simple (e.g., trivially easy for a computer program to do; Newell et al., 1963).

Curiously, certain versions of the selection task eventually emerged on which people did quite well, even as they continued to perform poorly on the original version of the task. One of the most well known of these tasks which elicit good reasoning performance is the “drinking age problem” (Griggs and Cox, 1982), in which the conditional rule is *If a person is drinking beer, then they must be over 21”* (with the card options thus being: *Drinking beer, Drinking soda, 17 years old,* and *22 years old*). These content-based effects on reasoning bedeviled researchers and led some of them to seek out new theories and criteria by which to evaluate human reasoning abilities.

Human conditional reasoning does not follow the normative model of formal logic. But performance on Wason’s selection task can be analyzed in terms of *other* normative models also (Elqayam and Evans, 2011). One can use deontic logic (conditional rules that regulate permissions and obligations) to evaluate correct versus incorrect responses. Cheng and Holyoak (1985), Holyoak and Cheng (1995) proposed that people induce pragmatic reasoning schemas, which closely parallel deontic logic principles, based on past experiences. Cosmides (1989), Cosmides and Tooby (1992) developed an explanation for selection task content effects based on evolved adaptations for reasoning about social contracts (conditional rules about reciprocal altruism, such as *If you take the benefit, then you must pay the associated cost*). One can alternatively use Bayesian reasoning or probability theory (Kirby, 1994; Oaksford and Chater, 1994, 1996, 2003; Oaksford et al., 1997) to evaluate correct versus incorrect responses in the selection task. In these models, correct responses are the selections which yield the highest expected information gain, whereas incorrect responses are those which yield little or no expected information gain. Finally, one can apply relevance theory (Girotto et al., 2001) to the selection task, proposing that the correct cards to select are the ones which are judged as most relevant to the current context.

This very cursory review of theories regarding human conditional reasoning illustrates a fundamental issue in terms of normative models in the study of human thinking. Most of these theoretical models of human reasoning aspire to be the one, best account of how human reasoning works. Researchers pit the models against each other, attempt to tally which model has the most support, best support, largest number of adherents, and so on. Which normative standard is the correct one? Which is correct at least in the case of human conditional reasoning? Apart from traditional disciplinary boundaries and preferences (or perhaps within-research laboratory traditions) there are no *a priori* justifications for these normative models. (And keep in mind that this illustration is just regarding conditional reasoning; it by no means exhausts the range of normative models for a realm as broad as “thinking.”) The situation – the existence of content effects, the proliferation of normative-based models, the ongoing lack of consilience – points to there being no general normative models for all of human thought. One possible reaction is to largely abandon normativism (Elqayam and Evans, 2011). Another approach, which is taken here, is to recognize that there are different domains with different normative standards. Continuing to search for one normative model to rule all of human thought, or even all of human reasoning, is untenable and needs to change.

## TIME SCALES AND RATIONALITY

There are several directions from which one can identify problems with the idea of general normative standards for rationality and human thought. Another aspect of this problem is illustrated by the tale of the village idiot:

Once upon a time there was a village idiot who, when offered a choice between a dime and a nickel, would invariably choose the nickel. Everyone would laugh at the stupidity of the village idiot, and then go back to their chores until the next time they felt like a laugh. This went on for many years, during which the village idiot repeatedly and reliably chose to take a nickel over a dime. One day, a kind-hearted person tried to explain the situation to the village idiot. “Look, even though a nickel is larger than a dime, it is only worth half as much. So you should choose the dime.” The idiot replied, “I know that. But if I choose the dime, people would stop offering me the choice between taking a nickel or a dime, wouldn’t they? Who would be that stupid?”

The implicit normative standard that underlies this story is a standard economic utility model: people are rationally self-interested and should prefer a larger quantity of a desired item over a smaller quantity (Marshall, 1920). What the not-quite-such-an-idiot village idiot had done, however, was realize that there is always an implicit time scale when considering the utility of a sequence of events. A very small time scale, capturing just one event, can indicate one behavior as having the highest overall utility (a dime is better than a nickel). A different, longer, time scale, though (say, capturing at least three choices), can indicate a completely different behavior as having the highest overall utility.

The tale of the village idiot can be understood as parallel to the distinction between a one-shot prisoner’s dilemma and a repeated prisoner’s dilemma (Axelrod and Hamilton, 1981). The prisoner’s dilemma is an economics game in which two people must decide whether to cooperate with the other person or defect against the other person. Mutual cooperation is rewarded, but not as much as defection when the other person cooperates (the “temptation payoff”). However, mutual defection does not pay as well as mutual cooperation, and cooperation when the other person defects yields a negative payoff (the “sucker’s payoff”). A one-shot prisoner’s dilemma has this payoff schedule and each person makes just one choice. In this one-shot version of the game the best strategy for each player is to defect, rather than cooperate, with the other player. As with the tale of the village idiot, this is based on the idea of utility maximization (in this case, maximization of the payoffs for each player) with a very small time scale of one move. Each player should go for the largest payoff (defecting), which also protects them from the worst outcome (being a sucker). If, however, the prisoner’s dilemma is played repeatedly between two players (also called an iterated game), there are strategies which are superior to constant defection in the longer time window. The most well known of these strategies is tit-for-tat, in which a player initially cooperates and then mirrors back whatever the previous choice was of the other player. Thus, two players can obtain the more modest (per play) reward of mutual cooperation rather than becoming stuck in mutual defection. These modest reward are *repeated* over the multiple rounds of the game. So, like the village idiot, each player accumulates multiple smaller payoffs which sum up to a much larger overall result than a single large payoff.

The effects of different time scale references also maps onto the idea of reciprocal altruism (Trivers, 1971) as a solution to the “problem of altruism” in biology. As evolutionary biologists considered the implications of evolutionary theory for the behavior of organisms, they realized that there seemed to be an overarching principle of complete self-interest: an individual should be focused intently on passing *their* genes into future generations and not at all interested – if anything, be antagonistic toward – other individuals managing to get their competing genes also into future generations. Yet in many cases animals did things which appeared to *help* other individuals, at a cost to themselves, which seemed to directly contradict the evolutionary theory implications. Hence, the “problem” of altruism. Along with the idea of kin-based altruism (Hamilton, 1964), a major explanation of these anomalous altruistic behaviors is the idea of reciprocal altruism (Trivers, 1971). The key insight for reciprocal altruism is that a single act of altruism (like cooperating in the prisoner’s dilemma) can make sense if there is a reciprocal act of altruism with the roles reversed. So long as the value of the help experienced by each recipient is greater than their experienced cost of helping, there is a resulting net gain for both parties (known in economics as “gains in trade”). Once more, part of the key insight is to consider multiple, reciprocal behaviors between the two individuals – an expanded window of time rather than a thin slice.

## DEEP RATIONALITY AND HUMAN THOUGHT

*How can they say my life is not a success? Have I not for more than 60 years got enough to eat and escaped being eaten?*

Smith (1931)

A resolution exists to this situation of arbitrarily conflicting normative models, many of which neglect the role of longer time scales, and it has been most fully and recently articulated by Griskevicius and Kenrick (2013), Kenrick and Griskevicius (2013), Kenrick et al. (2009), Kenrick et al. (2012). This resolution begins with a concept of “deep rationality,” which presumes that rationality must be defined with respect to a very long time frame: the evolutionary selection pressures which shaped the human mind. There have been a multitude of different selection pressures and this insight, together with the idea of cognitive modularity, leads to the idea that there never was (and never will be) a single, proximate standard for normative rationality. Instead there are multiple motives which every person is balancing at any given time. In other words, to the extent that there is any overarching standard of rationality that designed our minds, it is not “don’t screw up” but rather “survive and reproduce.” This ultimate criteria, however, is not immediately useful beyond its ability to frame more specific problem solving domains (also see Buss, 1995; on top-level versus mid-level evolutionary theories). Rather than a general “survival and reproduction” criterion, this model presumes that there are different standards for a successful decision in different social problem domains such as: self-protection, status, affiliation, mate acquisition, mate retention, kin care, and disease avoidance. These domains provide fundamental motivational goals for people, but because there are several of them we can conceptualize our minds has having a number of different “subselves,” each with different motives, different decision making processes, and even (from a more domain-general perspective) different cognitive biases.

Because different adaptive problems require different “rational” solutions, these solutions can only ever obey a local normative model which will inevitably break down once the topic under evaluation moves too far afield from the particular domain which constituted the evolutionary selection pressure and adaptive problem which created it (see also, Sperber, 1994 on the idea of proper domains for evolved mechanisms versus actual and cultural domains of application). Evolution designed many different cognitive programs, each embodying particular logics, designed to function well in particular contexts. In other words, the domain specificity of the cognitive mechanisms in the human mind implies that not only is there empirically no single normative standard of rationality which works across all of human cognition, but that there are good theoretical reasons why we should *expect* this to be the case.

This perspective belies many of the traditional criteria for normative models of rational though, such as obeying transitivity or the conjunction rule in probability; these are specifically applied as abstract, content-independent, and domain-general criteria. We should, in fact, be completely and utterly unsurprised that these types of criteria fail when they are applied to domains in which they do not correspond to the decision making adaptations evolution built within those domains. The fact that different theoretical models of conditional reasoning, as outlined above, each work particularly well within the context of particular reasoning contents should be alerting us to the fact that there is no one “human reasoning” normative model. Instead there are many cognitive mechanisms, each tailored to help us reason in an adaptive way about many different types of situations. It is even plausible that some limited abilities are included in this menagerie that enable general, abstract reasoning abilities when none of the specialized, evolutionarily-relevant contexts apply.

Or consider the prisoner’s dilemma described earlier. Not only does using a different time scale change the nature of this dilemma, but specifying different players in the dilemma can change it as well. The classic prisoner’s dilemma is played by two strangers (despite the allegorical “prisoners” being almost certainly friends). Strangers playing each other in the dilemma helps us to consider the situation more clearly in terms of domain-general, abstract rationality. But if, for instance, the prisoner’s dilemma is played between biologically related individuals (kin), then issues of kinship and kin selection (Hamilton, 1964) come into play. The payoffs within a prisoner’s dilemma are fundamentally altered when the genetic fitness implications of playing with kin are factored into them: the points that a genetically related opponent obtains in a prisoner’s dilemma are implicitly benefiting one’s own biological fitness as well (due to the proportion of genes shared by virtue of common descent). For close kin, in fact, the dilemma actually resolves itself and there can be a mutually optimal equilibrium state (Kenrick et al., 2008, 2012). Strangers playing against each other in a prisoner’s dilemma serves to simplify the situation, but it also is makes the situation less ecologically realistic; most of our real world interactions are with family, friends, and acquaintances.

So should “deep” evolutionary rationality serve as the definitive normative standard of behavior? Not necessarily. It is still critical to remember that human behavior has a foundation in cognitive adaptations, built by evolution over previous generations, and then further developed and filtered through our own experiences. A set of individual behaviors, within specific situations, can violate deep rationality, and violate it as a normative standard or as a descriptive standard. Being deeply rational is not the same as being omnipotent or omniscient. We are executers of cognitive programs (our evolved, mental adaptations). This means that there will be certain types of situations in which the cognitive programs produce “wrong” responses. One type of such situations is when there is an environmental mismatch: the responses which were shaped by many prior generations of evolution are no longer the best responses in our modern environment (e.g., our strong preferences for fats and sugars even when we already have enough; our general lack of desire for fiber in our diets even when we are in need of it). Another type of situation in which individual behaviors, based in deep rationality, can appear to be in violation of any normative or descriptive model is when there is a probabilistic outcome which is driving the selection pressure for that behavior (e.g., adolescent risk taking can appear to be irrational because it leads to some injuries and deaths, but at the same time if those behaviors produced an even larger social status and reputation benefit for the more successful risk takers then the overall behavioral tendency can be positively selected for nonetheless).

## EXAMPLES

*Having a child is surely the most beautifully irrational act that two people in love can commit*.

Cosby (1987)

A couple of examples may help clarify the implications of taking a “deep rationality” perspective within a modular mind. The decision to have a child or not has been characterized as fundamentally sound (e.g., Holm, 2005), fundamentally unsound (e.g., Häyry, 2005), and even fundamentally impossible to evaluate (Paul, 2015 forthcoming). Certainly an economic cost/benefit analysis in modern environments does not support the position that having children is a rational choice. (The U.S. Department of Agriculture estimates that the cost of raising a child to the age of 17 is $269,520 (for families making over $70,200 per year.) On the other hand, a biological analysis would point out that reproduction is the most fundamental purpose of living organisms, and therefore any price is worth paying to have children. Somewhere in between these radical extremes are real people, who very often do opt to have children yet who also nearly always limit their reproductive rate to something significantly less than what they would theoretically accomplish if they devoted all their resources to having children. Brase and Brase (2012) found that both men and women have strong, emotional reactions (both positive and negative) to the prospect of having children, suggesting that there are countervailing forces at work in people’s decisions about having children.

One compelling way to make sense of all these conflicting ideas and outcomes is to realize that desires to have children is but one of several different fundamental motives residing in people. We want to have children. But we also want to be safe (self-protection), we want to be respected (status), we want to be part of larger social groups (affiliation), we want to have and keep sexual partners (mate acquisition, mate retention), and we want to be healthy (disease avoidance).

Greed, for lack of a better word, is good. Greed is right, greed works.

[Gordon Gekko (Pressman and Stone, 1987)]

Now, a counterexample. The financial markets are perhaps the most elevated bastion of true and complete rationality. Adam Smith’s “invisible hand” (Smith, 1776) rests on the idea of everyone acting in their rational economic self interests, and many people consider the Western financial markets to be a huge success of modern society. A closer look at the underlying foundations and assumptions of the modern financial market, however, can illustrate how its success arrives by stripping out all but one fundamental motive. The financial markets are not (or are minimally) interested in self-protection, status, affiliation, mate acquisition and retention, kinship, or disease avoidance. The financial markets are about money. With just one, clear motivating goal, it becomes possible to be completely rational in relation to the accomplishment of that goal. Critics of how the financial markets operate will often note, in various ways, this issue. Concerns include problems with the ethics of the financial markets, the effects of modern economic practices on human safety, security, or happiness. But these concerns are tangential to the central goal of the financial markets, so they form only externally imposed borders on behavior (e.g., through government regulations of disallowed actions).

If former presidential candidate Mitt Romney is correct that “corporations are people” (Rucker, 2011), what type of people are they? They are people who exist largely within the world of modern financial markets, and they therefore live lives that are single-mindedly about financial self-interest. Without all the other fundamental concerns that regular people have about their relationships with fellow humans, they quite possibly also qualify as psychopaths (Achbar et al., 2003). Before thinking that I am particularly anti-corporation, please note that it is also true that corporations are, by design and by law, exactly this way because we as a society have chosen to make them that way. Corporations cannot do anything other than act purely in their complete economic self interest. (Interesting things also, of course, occur due to the fact that corporations are often managed by regular humans who *do* recognize a multitude of other fundamental motives, and these corporation owners can elect to make decisions based on their other motives, sometimes with the approval of shareholders and sometimes without).

## CONCLUSION

This special topic in *Frontiers* (in which this article appears) describes the evolutionary approach to studying human thinking as *empirical normativism* in which human thinking is considered correct because it is the thinking which occurs (i.e., that there is no external evaluative standard). Such a view is described as a Panglossian framework, in which human thinking is considered *a priori* as being rational. This is unfair and incorrect.

First of all, to the extent that anyone actually exists who could be considered a Panglossian, this framework has never distinguished the evolutionary approach. This caricature of adaptationism is trafficked often by its critics and repeated by many who hear this criticism without realizing that it has been debunked repeatedly and by multiple, independent evaluations (e.g., Tooby and Cosmides, 1992; Borgia, 1994; Queller, 1995; Buss et al., 1998). Second, adaptationist models are not empirically driven, but rather based on evolutionary principles. The hypotheses (which are normative, in the sense of making predictions about what ought to happen, if the theory is correct) are based on careful consideration of evolutionary selection pressures, the constraints faced by a particular species, and existing evidence. Third, the appropriate issue is therefore not empirical normativism versus prescriptive normativism (which evaluates human thinking based on externally imposed criteria such as logic or probability theory), but rather how one should construct normative models of human thinking. Is it more useful to work with proximate models of normative rationality which proliferate under the traditional prescriptive normativism framework; models which becomes problematic as they struggle to accommodate *ad hoc*, competing domains of application? Or, is it better to work with higher level models of rationality, based on an evolutionary understanding of the central problems the mind has been sculpted to address?

An evolutionary framework, as outlined here, indicates that there are some normative standards which are useful for understanding the nature of human thinking, but that those standards are different from many of the normative standards proposed by prescriptive normativism. The search for a single normative model for all of human thinking is futile, because the multiple selection pressures which shaped the mind led to multiple cognitive mechanisms. A large-scale modularity of thinking processes is required, and in fact points toward useful ways to escape the multitudes of single-model theories which often stand in stalemates against each other. One specific version of this evolutionary modularity approach is the model of deep rationality (Kenrick et al., 2009, 2012), which specifies a set of fundamental motivational goals, each of which entails distinct patterns of reasoning and thinking (and which may be consistent, inconsistent, or orthogonal to each other).

## Conflict of Interest Statement

The author declares that the research was conducted in the absence of any commercial or financial relationships that could be construed as a potential conflict of interest.
